# COVID-19: High-JAKing of the Inflammatory “Flight” by Ruxolitinib to Avoid the Cytokine Storm

**DOI:** 10.3389/fonc.2020.599502

**Published:** 2021-01-08

**Authors:** Cirino Botta, Alessia Indrieri, Eugenio Garofalo, Flavia Biamonte, Andrea Bruni, Pino Pasqua, Francesco Cesario, Francesco Saverio Costanzo, Federico Longhini, Francesco Mendicino

**Affiliations:** ^1^ Hematology Unit, Department of Hemato-Oncology, “Annunziata” Hospital of Cosenza, Cosenza, Italy; ^2^ Telethon Institute of Genetics and Medicine (TIGEM), Pozzuoli, Italy; ^3^ Institute for Genetic and Biomedical Research (IRGB), National Research Council (CNR), Milan, Italy; ^4^ Anesthesia and Intensive Care Unit, Department of Medical and Surgical Sciences, “Magna Graecia” University, Catanzaro, Italy; ^5^ Department of Clinical and Experimental Medicine, “Magna Graecia” University, Catanzaro, Italy; ^6^ Anesthesia and Intensive Care Unit, “Annunziata” Hospital of Cosenza, Cosenza, Italy

**Keywords:** COVID-19, ruxolitinib, hyperinflammation, ferritin, JAK2

## Abstract

Since SARS-CoV-2 outbreak in December 2019, world health-system has been severely impacted with increased hospitalization, Intensive-Care-Unit (ICU) access and high mortality rates, mostly due to severe acute respiratory failure and multi-organ failure. Excessive and uncontrolled release of proinflammatory cytokines (cytokine release/storm syndrome, CRS) have been linked to the development of these events. The recent advancements of immunotherapy for the treatment of hematologic and solid tumors shed light on many of the molecular mechanisms underlying this phenomenon, thus rendering desirable a multidisciplinary approach to improve COVID-19 patients’ outcome. Indeed, currently available therapeutic-strategies to overcome CRS, should be urgently evaluated for their capability of reducing COVID-19 mortality. Notably, COVID-19 shares different pathogenic aspects with acute graft-versus-host-disease (aGVHD), hemophagocytic-lymphohistiocytosis (HLH), myelofibrosis, and CAR-T-associated CRS. Specifically, similarly to aGVHD, an induced tissue damage (caused by the virus) leads to increased cytokine release (TNFα and IL-6) which in turn leads to exaggerated dendritic cells, macrophages (like in HLH) and lymphocytes (as in CAR-T) activation, immune-cells migration, and tissue-damage (including late-stage fibrosis, similar to myelofibrosis). Janus Kinase (JAK) signaling represents a molecular hub linking all these events, rendering JAK-inhibitors suitable to limit deleterious effects of an overwhelming inflammatory-response. Accordingly, ruxolitinib is the only selective JAK1 and JAK2-inhibitor approved for the treatment of myelofibrosis and aGVHD. Here, we discuss, from a molecular and hematological point of view, the rationale for targeting JAK signaling in the management of COVID-19 patients and report the clinical results of a patient admitted to ICU among the firsts to be treated with ruxolitinib in Italy.

## Introduction

Since late December 2019, severe atypical pneumonia cases requiring prompt hospitalization and frequent access to Intensive care Units (ICUs) have been reported in China. The etiological microbial agent was identified as a novel member of the β-Coronaviridiae family, named SARS-CoV-2 (1). The new epidemy [declared as pandemic since March 12^th^, 2020 by WHO (2)], rapidly spread in and out of the country, involving millions of cases around the world [https://www.ecdc.europa.eu/en/geographical-distribution-2019-ncov-cases]. Early data from Chinese studies claimed an overall case-fatality rate of about 2.3% (up to 14.8% in patients aged >80 years) (3). However, the infection has shown a more aggressive clinical course in European countries and USA, with an overall mortality rates of about 10% (4), which increases to 26% in ICU-admitted patients, being even higher in the elderly population and in patients with pre-existing comorbidities (hypertension, diabetes, obesity) (5, 6). Clinically, SARS-CoV-2 related disease (COVID-19) ranges from asymptomatic or mild/moderate symptoms (fever, arthro-myalgia, nausea, and diarrhea, anosmia) to a severe respiratory illness requiring ventilatory support and, in a small percentage of patients, extracorporeal membrane oxygenation (ECMO) (6, 7). Overall, approximately 20% of patients deteriorate (often rapidly) about 7-10 days after the onset of symptoms (8) and about 25% will require mechanical ventilation (associated with increased mortality risk) (8). Although the exact mechanism of lung damage is still under investigation, some Chinese reports and previous experience with SARS/MERS related diseases (9–12) focus on the possibility that SARS-CoV-2 induces alveolar macrophages activation and release of inflammatory cytokines and chemokines (i.e., cytokine release syndrome, CRS), that further recruit innate (monocytes and neutrophils) and adaptive (T and B cells) effectors to the lung. This event likely promotes an inflammatory cascade at the tissue site that in turn, induces thromboembolic events and a condition of acute respiratory failure (ARDS-*like*). In some cases, CRS related damage extends to liver, heart, and kidney, leading to multiorgan failure (13, 14) and/or macrophage activation syndrome (MAS) (15, 16). The most common clinical manifestation, viral pneumonia, affect more than 90% of symptomatic patients within 4 days from onset (17). However, in severe patients, a progressive loss of epithelial-endothelial integrity with capillary damage, neutrophils, and complement activation with localized intravascular coagulation could be observed (18). These pathological findings suggest that this most advanced and potentially fatal stage (19, 20) relies on the (over-) involvement of the adaptive immunity more than on a direct effect of the virus on the lung epithelium.

**Figure 1:**
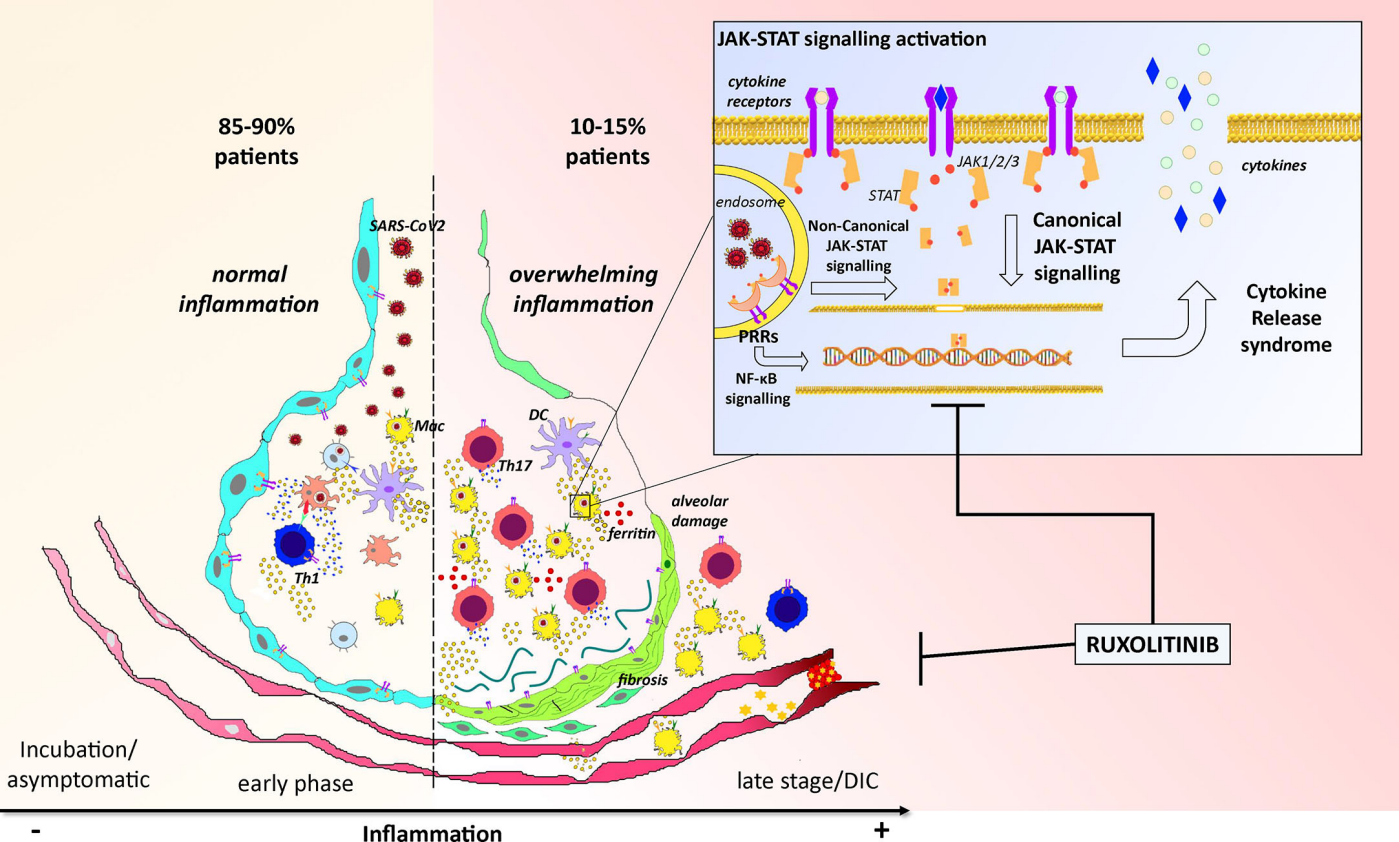
Cartoon representing the overview of the pathogenesis of COVID-19 and the potential activity of ruxolitinib. The left part of the picture reports the most common pathogenetic events happening along SARS-Cov2 infection in 85/90% of patients: after virus invasion, antigen presentation, and establishment of an adaptive immune response, lungs reach the viral clearance with low or no symptoms. The right part of the figure reports instead the worse scenario where an exacerbation of the inflammatory response characterized by increased neutrophils and Th17 activation lead to ferritin overload, alveolar damage with fibrosis, and disseminated intravascular coagulation (DIC) which could potentially kill the patient. These events mainly depend on an uncontrolled activation of the JAK-STAT pathway (trough canonical and non-canonical signaling) which finally leads to uncoordinated production and release of inflammatory cytokines (CRS) within the alveolar microenvironment. By targeting the JAK-STAT pathway, ruxolitinib could disrupt this “vicious circle” and restore the correct alveolar functionality.

Currently, no specific treatments exist for COVID-19 and many drugs such as lopinavir/ritonavir (21–27), remdesivir (28, 29), chloroquine (30), or hydroxychloroquine (31), azithromycin (32, 33) as well as the anti- IL-6 receptor monoclonal antibody tocilizumab (34, 35), failed to demonstrate a clear clinical benefit. Here, we review the current knowledge on the immune and inflammatory response to SARS-CoV-2 from a hematological and molecular point of view (**Figure 1**) and we report the results of one of the first COVID-19 patients admitted to ICU treated with the JAK2-inhibitor ruxolitinib, belonging to a group of patients who started this treatment under off-label use (under physician responsibility) on March 27^th^ 2020.

## COVID-19: Immunological and Inflammatory Changes

Physiologically, the immune system responds to viral infection by activating both cellular and humoral responses. However, while a quick and coordinated immune response leads to rapid viral clearance, an overwhelming inflammatory response and uncontrolled adaptive immunity could be harmful for the host. As already stated, CRS (and its sequelae) is considered the main cause of COVID-19 patient’s death. Indeed, in patients admitted to ICU and with an extensive lung injury, proinflammatory cytokines and chemokines [such as IL2, IL7, TNFa, IL17, Monocyte Chemoattractant Protein-1 (MCP-1), and others] were found to be significantly upregulated (36, 37). While the triggers of CRS still remain to be completely elucidated, several mechanisms could be hypothesized for similarity with other diseases. Specifically, a damaged tissue (endothelial and alveolar epithelial cells) could lead to the production and release of i) chemotactic factors attracting monocytes and neutrophils and ii) specific molecules commonly known as damage-associated molecular patterns (DAMPs) such as high-mobility group box 1 (HMGB1), ATP, and cell free DNA. DAMPs, together with pathogen-associated molecular patterns (PAMPs), including viral RNA/DNA, are recognized by a group of receptors (Pattern Recognition Receptors, PRRs) such as toll-like, RIG-I-like, and NOD-like receptors, expressed on both macrophages and neutrophils, whose engagement activate a series of downstream regulators (NFKB, inflammasome, JAK/STAT) which induce proinflammatory cytokines and chemokines release (38). To be noted that ferritin, usually elevated in “hyperinflammed” COVID-19 patients, is overexpressed during CRS and released by tissue infiltrating macrophages further increasing local inflammation by working as proinflammatory molecule (see below).

By looking at the immunological side of COVID-19 infection, several recent studies identified lymphocytopenia, neutrophilia and neutrophil-to-lymphocyte ratio as hallmarks of worse prognosis (39). Overall, the lymphopenia affects both the T and B compartments. CD4 and CD8 T cell subpopulations where significantly reduced in absolute count, while a significant relative increase in the Th17 pro-inflammatory subpopulation was observed (40). The latter is known to be involved in the pathogenesis of autoimmune and cancer disease and rely on the microenvironmental presence of IL-6 and IL-23 (41, 42). Interestingly, it is thought that, in analogy so SARS-CoV, SARS-CoV2 could infect both lymphocytes and monocytes/macrophages through a still unknown mechanism (ACE2 is present at a very low level on the surface of these cells). This event could lead to a further exacerbation of the inflammatory machinery due to the fact that viral component (RNA) could be sensed by intracellular PRRs. Additionally, the observed increase in Naïve/memory ratio within T cell population, coupled with an unexpected reduction in Tregs, has been hypothesized to further activate systemic inflammatory response (41, 42).

Regarding the myeloid compartment, in a recently published single cell RNAseq study, IL1b, accordingly to what already stated, emerged as a new hallmark of COVID-19 infection. Specifically, it was found to be upregulated (as compared to control) in CD14+ monocytes and dendritic cells from COVID-19 patients, thus further supporting an excess in inflammatory response.

## Ferritin and COVID-19: The Influence of Iron Metabolism in Inflammatory Response to the New Coronavirus

As previously stated, high levels of serum ferritin have been found to be a risk factor for COVID-19 severity and assessing this serum biomarker during hospitalization could be of utmost importance to identify high-risk patients with COVID-19 (43–47). In agreement, patients with diabetes, which faced a higher probability to experience complications from COVID-19, exhibited elevated serum ferritin levels upon hospitalization (48). Furthermore, autoptic studies on SARS-CoV-2 patients revealed elevated ferritin levels (49). The exact mechanism underlying the association of hyperferritinemia and COVID-19 severity is still under active investigation, but several reports highlighted an increased frequency of bilateral pulmonary infiltration and concurrent coagulopathy in patients with hyperferritinemia (≥500 ug/L) (43–47).

Ferritin is a major intracellular iron storage protein (50) and its accumulation (hyperferritinemia) is a hallmark of the so-called anemia of inflammation (AI), which is common in patients bearing a prolonged immune activation, typical of malignancies, infections, autoimmune diseases, and chronic kidney or pulmonary diseases (51).

The origin of circulating serum ferritin during inflammatory conditions is still not clear. Additionally, increasing evidence supports the concept of ferritin as a modulator of systemic and local inflammation (52). Indeed, despite most of the serum ferritin derives from tissue injury and, in particular, from hepatic cells death, during “hyperferritinemic syndromes”, ferritin could be even actively released by hepatocytes (53) as well as by macrophages (54). Once released, ferritin loses part of the iron content determining extremely high serum levels of “free iron”, which, in turn, can deteriorate the inflammatory reaction by inducing a marked pro-coagulant state (55). “Free iron” may, indeed, favor the production of reactive oxygen species (ROS) (55) and promote oxidative stress on red blood cells and fibrin activation, thus leading to the production of dense clots, involved in stroke pathogenesis (56). Due to the property of iron chelation to modulate the inflammatory response through the reduction of ROS production, the activity of this therapeutic approach in patients with SARS-CoV-2 infection has been recently investigated (57). However, further studies are needed to confirm the role of serum ferritin as a therapeutic target as well as predictive/prognostic marker of COVID-19 patients’ outcome.

## COVID-19 Hyperinflammation: Molecular Mechanisms and Targeting Opportunities

The identification of the intracellular signaling pathways underlying host immune systems response may be crucial for the treatment of COVID-19 since it can lead to the identification of new therapeutic actionable targets. Notably targeting intracellular molecules rather than viral proteins shows the advantage to exert less-selective pressure on viral populations, being less likely to be “escaped” by virus mutations.

As described before, upon binding of the viral “spike” protein to the target cell by the ACE2 receptor, viral RNAs are detected by the PRRs, that, in turn, activate different downstream transduction pathways crucial for the proper antiviral response (58). Among them, NF-κB and JAK/STAT, key molecular pathways in the immune response, raised a particular interest representing attractive targets for therapeutic intervention (59).

The JAK/STAT signaling, in particular, is one of the main regulatory cell pathways that transduces extracellular signals in response to a variety of cytokines, lymphokines, and growth factors, and regulates key cellular processes such as differentiation, cell cycle, apoptosis and immune response (60, 61). The JAK non-receptor tyrosine kinase family is composed by Jak1, Jak2, Jak3, and Tyrosine kinase 2 (Tyk2) proteins. When extracellular signals are detected by a specific JAK-associated receptor, JAKs phosphorylate a member of STAT family (including STAT1, STAT2, STAT3, STAT4, STAT5a, STAT5b, and STAT6), which results in dimerization and translocation of STAT into the nucleus where it activates or suppresses the transcription of different genes involved in immune regulatory, differentiation, cell cycle, and apoptotic signaling (60, 61).

Interestingly, IL-6, reported to be increased in COVID-19 patients, is one of the major activators of JAK/STAT signaling (47, 62, 63). IL-6 activates JAK/STAT signaling in different cell types expressing IL-6 receptors, stimulating, in a positive feedback loop, IL-6 production, and release (42, 64). Aberrant activation of this pathway has been reported in patients affected by chronic inflammatory diseases (e.g., arthritis rheumatoid), and could occur in COVID-19 patients, thus exacerbating host inflammatory response. Of note, chronic increase of serum IL-6 levels has been associated with higher risk of cardiovascular events (65, 66), thus supporting its role in the development of cardiovascular complications (including inflammation-dependent diffuse microangiopathy with thrombosis) in COVID-19 patients. Interestingly, IL-6 synthesis and secretion could be induced by Angiotensin II (ATII), released by inflamed vessels, in a JAK/STAT-dependent manner. Specifically, ATII binding to ATII receptor type 1 (ATR1) activates JAK/STAT downstream signaling and promotes the production of IL-6 in a positive inflammatory feedback loop (67, 68). Notably, it has been shown that SARS-CoV spike could reduce ACE2 expression, resulting in the overproduction of ATII by the related enzyme ACE (69, 70). Accordingly, it could be hypothesized that SARS-CoV-2 acts in a similar manner by: 1) inducing an overproduction of ATII, which 2) enhances IL-6 production in a ATR1/JAK/STAT-mediated manner that 3) finally leads to inflammation-dependent microangiopathy and lung injury.

Based on these findings, approved drugs inhibiting IL-6/JAK/STAT signaling may represent a valuable tool in the treatment of COVID-19. In particular, as already reported, drugs as Tocilizumab, have been investigated in COVID-19 with contrasting results. About 40 clinical trials are ongoing to test tocilizumab, alone or in combinations, in patients with COVID-19 (clinicaltrials.gov and clinicaltrialsregister.eu).

On the other hand, JAK signaling inhibitors (baricitinib, fedratinib, and ruxolitinib), already approved for the treatment of several diseases including rheumatoid arthritis, myelofibrosis and acute graft-versus-host disease (a-GVHD), have been reported to counteract the host inflammatory response dependent on excessive pro-inflammatory cytokines and chemokines release, thus representing an interesting drug repurposing therapeutic strategy (see below). On these premises, a number of clinical trials are investigating the efficacy and safety of JAK inhibitors in COVID-19 patients, especially taking into account the balance between benefits and potential side effects connected to these treatments (**Supplementary Table 1**).

## Targeting the JAK Signaling: The Promise of Ruxolitinib

Ruxolitinib is a JAK inhibitor currently approved for the treatment of JAK-STAT dependent myeloproliferative syndromes (MFI, in both USA and Europe) and graft-versus-host disease (USA). As previously explained, due to its mechanism of action, the drug presents potent immunosuppressive and anti-inflammatory properties on both innate (dendritic cells, macrophages, and neutrophils) and adaptive (T cells) immune effectors (71–74) and reduces the secretion of several pro-inflammatory mediators including IL-6 and TNF-alpha (75). Additionally, thanks to it safety profile, ruxolitinib has been shown to be suitable even for elderly population with myelofibrosis (76).

Ruxolitinib demonstrated anti-inflammatory and immunomodulatory activity in hemophagocytic lymphohistiocytosis (HLH) and steroid refractory aGVHD (both resembling many characteristics of the inflammatory response against SARS-Cov-2) where induced a strong reduction of ferritin and LDH and of other inflammatory molecules such as IL-1, TNF, and MIP1a (77), coupled with a recover from T and B cell lymphopenia and the normalization of CD4/CD8 ratio (78). These data support the potential role for ruxolitinib, as a drug repurposing strategy for the treatment of SARS-CoV-2 driven CRS syndrome.

However, some specific concerns should be taken into account: as other JAK inhibitors, ruxolitinib impairs the capability of antigen presenting cells (such as macrophages and dendritic cells) to produce, among others, type I interferons, Il-12, IL-15, and IL-23. This event negatively influences NK activation, antigen presentation and Th1/Th17 polarization, with consequent alterations in antigen-specific T cells response, including viral clearance, providing a possible mechanistic explanation for the increase of infection rates in patients with MPN undergoing long-term ruxolitinib treatment (36, 79–87).

Along this line, it could be conceivable that a short-term treatment with ruxolitinib could be suitable to abrogate CRS response during viral infection and avoididing the risk of innate and adaptive systemic anti-viral response impairment. On these bases, we requested and obtained the approval for using ruxolitinib in a patient with COVID-19 admitted to ICU, *via* nasogastric tube. We describe below the results obtained in this seriously ill patient.

## From Theory to Clinical Practice: Case Report of the First ICU Patient Treated With Ruxolitinib

**Figure 2:**
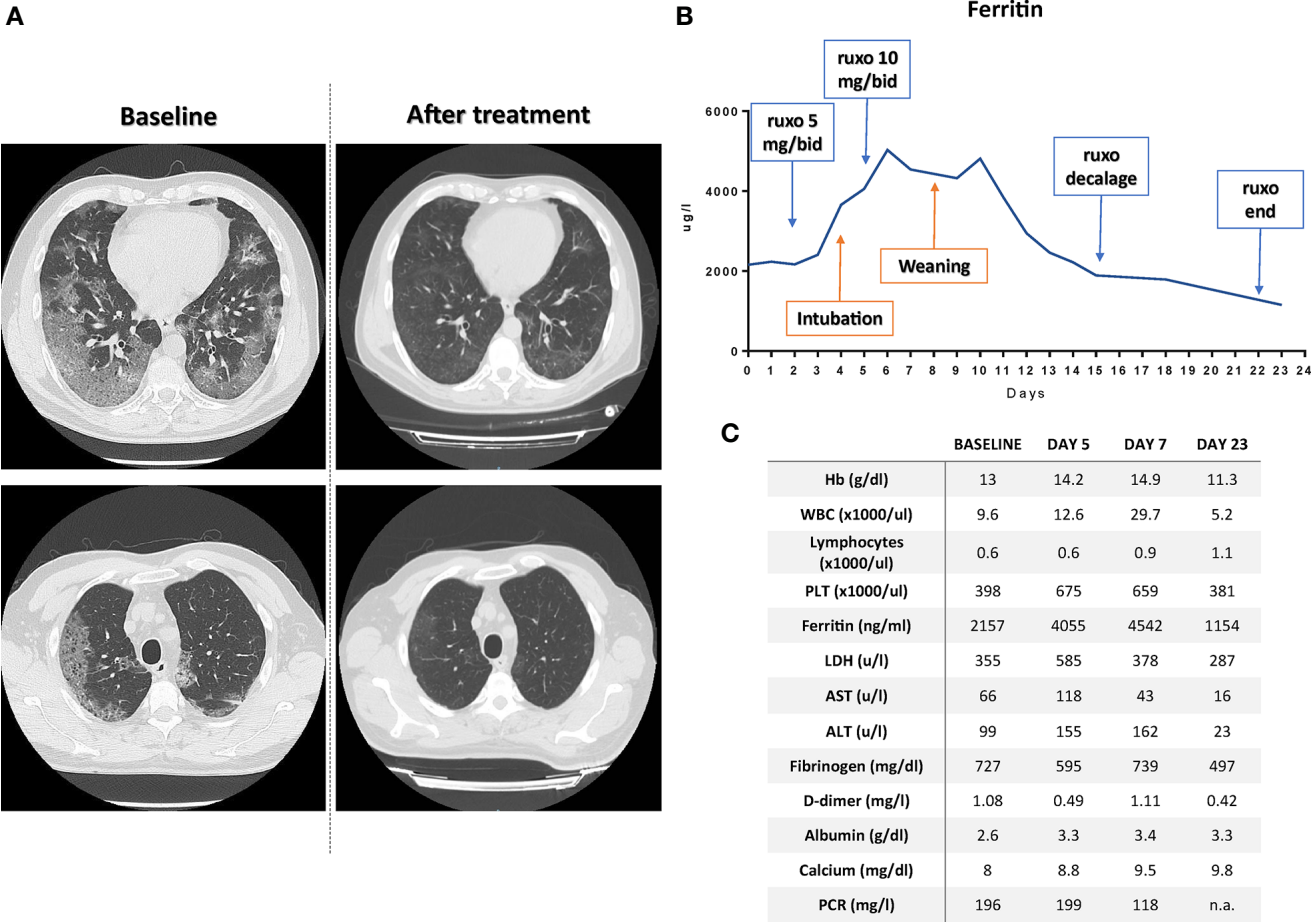
**(A)** CT-scan at baseline and after ruxolitinib treatment demonstrating the resolution of the ground-glasses areas at two different levels. **(B)** Timeline reporting ferritin modulation according to patient treatment; ruxo, ruxolitinib. **(C)** Patient laboratory values at baseline, 5 and 7 days, and at the time of discharge. n.a., not available.

In middle March 2020, a 54-year-old man accessed the emergency unit presenting with fever poorly responsive to acetaminophen and antibiotic therapy with azithromycin. After about 10 days from symptoms appearance and taking into account the progressive worsening of respiratory function, a nasal and pharyngeal swab was carried out for the research of SARS-COV-2 genes, confirming the diagnosis of COVID-19. The baseline high resolution thoracic CT scan (**Figure 2A**) detected signs of lung disease such as multiple bilateral “frosted glass” areas and interstice thickening. Due to worsening in respiratory function, the patient began a Venturi mask respiratory support quickly replaced by a C-PAP application due to insufficient peripheral oxygenation. Since hospital admission, the patient began the standard medical therapy used at that time in our institution, which included a combination of hydroxychloroquine, azithromycin, lopinavir/ritonavir, corticosteroids and LMWH, without significant clinical benefit after 10 days of continuous administration. Indeed, inflammatory markers such as LDH, ferritin, CRP, and IL-6 constantly increased, and the worsening of respiratory function (PaO2 FiO2 ratio < 200) required ICU admission and orotracheal intubation with passive mechanical ventilation. Continuous administration of neuromuscular blocker agents was started. Because of severe hypotension, continuous infusion of norepinephrine administration was necessary. Two days before ICU admission, taking into account age, systemic inflammatory status, radiological scenario, and clinical worsening, after signing an informed consent, the patient started a short-term treatment with ruxolitinib under a specifically authorized off-label access (the patient belongs to a group of patients for whom we asked authorization on March 23th 2020), granted by the Italian ethical committee for COVID-19 experimentation located at the National Institute for Infectious Diseases “Lazzaro Spallanzani” in Rome, under the complete responsibility of the physicians who requested the drug. At that moment, no shared and uniform criteria for patient selection were available, so, taking into account the activity of the drug, we chose to treat “hyperinflamed” patients only, defined as having 2 or more of the following criteria: ferritin> 400 ng/ml, lactate dehydrogenase (LDH)> 480 U/l, lymphopenia (lymphocyte count <1,000/ul), reactive protein C (CRP)> 5 mg/l, fibrinogen <200 mg/dl, albumin <3.9 g/dl, triglycerides> 150 mg/dl, aspartate aminotransferase (AST) or alanine aminotransferase (ALT)> 40 IU/l. Of note, mechanical respiration was not an exclusion criteria. As per hematological clinical practice, before treatment start, the patient was screened for HBV, HCV, and HIV positivity and for active or latent tuberculosis, also in consideration of the recent hypothesis that SARS-Cov-2 infections can cause lung inflammation leading to the reactivation of dormant tuberculosis in the lung (https://www.biorxiv.org/content/10.1101/2020.05.06.077883v1).

The treatment schedule was *ad hoc* designed, mimicking aGVHD therapy and included a 3-days “induction” phase (5 mg bid) followed by a “ramp-up” to 10 mg bid for 10 days and, lastly, a 7 days decalage phase (5 mg BID for 3 days followed by 4 days at 5 mg dose one once a day) to minimize the risk of serious adverse events due to “ruxolitinib withdrawal syndrome” (88).

Treatment with antiviral agents was continued, taking into account that in healthy subjects the concomitant administration of ruxolitinib with a CYP3A4 inhibitor resulted in an increase in Cmax and AUC by 33% and 91% (89), rendering unnecessary to increase ruxolitinib dosage over 10 mg bid. Twenty-four hours after increasing the dosage of ruxolitinib (administered through the nasogastric tube), we observed the first signs of improvement in the laboratory parameters (**Figures 2B, C** and **Supplementary Figure 1**), which allowed the start of weaning procedures on the 5th day from the start of the 10 mg bid therapy. After few hours, ventilation mode was shifted to Pressure Support Ventilation. The day after, because of the improvement of respiratory exchange, patient was extubated and not-invasive ventilation by helmet was performed. Overall, along recovery in ICU, patient underwent four pronation cycles and ruxolitinib administration continued after patient discharge in infectious disease department according to the treatment schedule. After 23 days from ruxolitinib initiation the patient was discharged at home in perfect condition and with all the laboratory parameters normalized (**Figure 2C**). Of note, post-treatment CT scan (**Figure 2A**) demonstrated clear resolution of lung disease without signs of interstitial fibrosis, a finding in line with the antifibrotic effect on bone marrow during ruxolitinib treatment in hematological diseases (90–97). The patient did not experience any side effect during the treatment, with the exception of a potentially treatment-related anemia (which spontaneously resolved after discharging, data not shown) (**Supplementary Figure 1**). The patient is currently (after 6 months from discharging) in perfect conditions with all laboratory exams within the range of normality (data not shown).

## Discussion

The cytokine storm in COVID-19 patients and the resulting hyperinflammatory syndrome with hyperferritinemia (36, 44, 98–107) often leads to the hypoxic lung lesions observed in ICU patients (36, 108, 109). Unfortunately, blocking interleukin-6 alone demonstrated to be rarely sufficient to counteract (or at least control) the establishment of the immune/inflammatory/thrombotic vicious circle (110, 111) responsible for patients’ death. In our view, a short-term inhibition of the JAK pathway could represent an important therapeutic weapon for these patients. Indeed, in addition to significantly reducing the serum levels of IL-6 and C-reactive protein (CRP) (77, 112), ruxolitinib could influence the regulation of several inflammatory cytokines (including IL-2, IL-5, and IL-10) (36, 113), and consequently reduce hyperferritinemia. The observed rapidity of action obtainable in just 2 h from administration (114) in modulation of the cytokine-induced STAT3 phosphorylation signal, and the possibility of administration by nasogastric tube, could make the drug definitely suitable for a therapeutic approach of emergency in serious ICU patients even in patients refractory to anti-IL-6 agents (115).

Taking into account that only 14% of COVID-19 patients develop symptoms that require hospitalization and oxygen support and that around 5% require ICU admission (36), strict criteria for patient selection should be used to identify hyperinflammed patients likely to benefit from ruxolitinib short-term administration (116). These “markers” should include advanced age, high SOFA score, D-Dimer values > 1 μg/L, and ferritin (44, 81, 117) and IL-6 values increase ​​(where and if dosage is possible). It is therefore clear that a close collaboration between emergency and infectious diseases physicians and hematologists (77, 78) is mandatory to render this treatment easy to manage and safe for patients. Being used as an oral treatment, under the supervision of hematologists, ruxolitinib could be administered at home in “hyperinflamed” patients with COVID-19 to avoid a worsening of clinical conditions and reduce the need for hospitalization, at an accessible drug cost per patient of about 3.500€ (price in Italy for the 5 mg 56 tablets package). Indeed, as shown in our case report, ruxolitinib could induce a strong anti-inflammatory response with normalization of different inflammatory parameters, quickly leading to a respiratory improvement.

On the bases of all biological premises (62, 115, 118–129), and of our preliminary results, we firmly believe that ruxolitinib presents a strong potential in overcoming lung and systemic complications caused by JAK/STAT-mediated immune hyperactivation during COVID-19 disease. Indeed, while recent works in this field (123–125, 127) demonstrated a promising activity of ruxolitinib in avoiding respiratory worsening and progression to mechanical ventilation in hyperinflamed patients at imminent risk to be admitted to ICU (by using different treatment schedules, including a dose escalation in case of not-responding patients), here we presented a case-report documenting the potential activity of the drug (with a slightly different 20-days schedule which include a preplanned dose intensification followed by a *decalage* phase) in patients already under mechanical ventilation, thus extending the possibility of using this drug in critical patients (our patient was indeed quickly intubated after treatment beginning and received ruxolitinib through a nasogastric tube). Anyway, while using different timings and schedules, all the studies reported a clinical benefit within few days from treatment starts without major signs of ruxolitinib-associated toxicities (mainly due to the short treatment courses) underscoring the need of larger studies (phase 3 studies are ongoing) to confirm the activity of the drug in hyperinflamed COVID-19 patients regardless of the respiratory support they need. Ruxolitinib-related side effects, when present, could be managed and resolved through a fruitful and humble cooperation between oncohematologists familiar to the drug (130–133) and clinicians from infectious disease, lung and intensive care units (118, 123–125, 127, 130, 134), without being misled by false convictions, lack of personal experience, overestimated toxicity (134) or by unethical conflicts of interests. In line with what recently highlighted in Lancet (135) and by La Rosée and colleagues (127), in this sad and difficult historical moment, patients deserve the best possible care and kind evaluation of new agents, communicating positive results immediately and promptly to the whole scientific community and translating new observational findings into structured (randomized) and methodologically correct clinical trials.

## Data Availability Statement

The raw data supporting the conclusions of this article will be made available by the authors, without undue reservation.

## Ethics Statement

The studies involving human participants were reviewed and approved by National Institute of Infectious Diseases Lazzaro Spallanzani’s Ethics Committee in Rome, Italy. The patients/participants provided their written informed consent to participate in this study.

## Author Contributions

CB and FM conceived and designed the paper. CB, FM, AI, FB, EG, and AB wrote the paper. FC supervised the infectious-diseases related part. FL, PP, and FSC supervised the whole paper development and provided suggestion for improvement. CB and FM equally contributed to the work and should be considered as co-first and co-last authors. All authors contributed to the article and approved the submitted version.

## Conflict of Interest

The authors declare that the research was conducted in the absence of any commercial or financial relationships that could be construed as a potential conflict of interest.
