# Supplementary figures and images for: COVID-19: High-JAKing of the Inflammatory “Flight” by Ruxolitinib to Avoid the Cytokine Storm
Source: Front Oncol. 2021 Jan 8;10:599502. doi: 10.3389/fonc.2020.599502 (PMC7819896; doi:10.3389/fonc.2020.599502)

Supplementary Fig 1

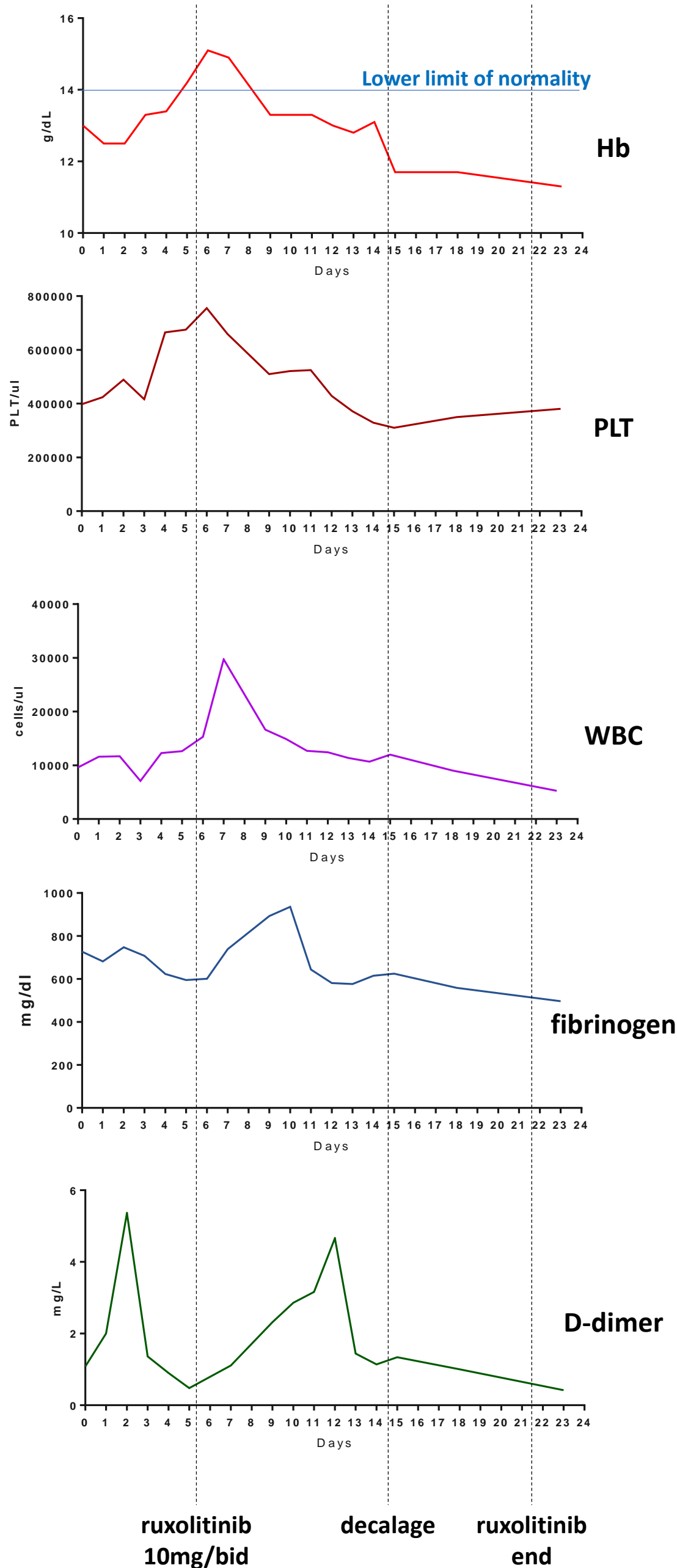

**Legend**  
**Hb:** hemoglobin  
**PLT:** platelets  
**WBC:** white blood cells

Supplement: Supplementary file 1 [file Image_1.pdf]
