# Supplementary material for: COVID-19: High-JAKing of the Inflammatory “Flight” by Ruxolitinib to Avoid the Cytokine Storm
Source: Front Oncol. 2021 Jan 8;10:599502. doi: 10.3389/fonc.2020.599502 (PMC7819896; doi:10.3389/fonc.2020.599502)
Supplement: Supplementary file 2 [file Table_1.docx]

| Row | Status | Study Title | Conditions | Study Results | Locations | Specifications |
| --- | --- | --- | --- | --- | --- | --- |
| 1 | Not yet recruiting | [Safety and Efficacy of Ruxolitinib for COVID-19](https://clinicaltrials.gov/ct2/show/NCT04348071?term=ruxolitinib&cond=Covid19&draw=2&rank=1) | COVID-19 | No Results Available |  | clinicaltrials.gov |
| 2 | Available | [Expanded Access Program of Ruxolitinib for the Emergency Treatment of Cytokine Storm From COVID-19 Infection](https://clinicaltrials.gov/ct2/show/NCT04355793?term=ruxolitinib&cond=Covid19&draw=2&rank=2) | COVID-19 | No Results Available |  | clinicaltrials.gov |
| 3 | Withdrawn | [Ruxolitinib to Combat COVID-19](https://clinicaltrials.gov/ct2/show/NCT04354714?term=ruxolitinib&cond=Covid19&draw=2&rank=3) | COVID-19 | No Results Available |  | clinicaltrials.gov |
| 4 | Not yet recruiting | [Ruxolitinib in the Treatment of Covid-19](https://clinicaltrials.gov/ct2/show/NCT04414098?term=ruxolitinib&cond=Covid19&draw=2&rank=4) | COVID-19 | No Results Available |  | clinicaltrials.gov |
| 5 | Recruiting | [Assessment of Efficacy and Safety of Ruxolitinib in Participants With COVID-19-Associated ARDS Who Require Mechanical Ventilation (RUXCOVID-DEVENT)](https://clinicaltrials.gov/ct2/show/NCT04377620?term=ruxolitinib&cond=Covid19&draw=2&rank=5) | COVID-19 | No Results Available | Georgetown University Hospital Washington, District of Columbia, United States  (and 8 more...) | clinicaltrials.gov |
| 6 | Recruiting | [Treatment of SARS Caused by COVID-19 With Ruxolitinib](https://clinicaltrials.gov/ct2/show/NCT04334044?term=ruxolitinib&cond=Covid19&draw=2&rank=6) | COVID-19  Severe Acute Respiratory Syndrome Coronavirus 2 | No Results Available | Grupo Cooperativo de Hemopatías Malignas Huixquilucan, Estado De México, Mexico | clinicaltrials.gov |
| 7 | Available | [Ruxolitinib Managed Access Program (MAP) for Patients Diagnosed With Severe/Very Severe COVID-19 Illness](https://clinicaltrials.gov/ct2/show/NCT04337359?term=ruxolitinib&cond=Covid19&draw=2&rank=7) | Severe/Very Severe COVID-19 Illness | No Results Available | Novartis Investigative Site Curug, Indonesia  Novartis Investigative Site Caserta, CE, Italy  (and 3 more...) | clinicaltrials.gov |
| 8 | Not yet recruiting | [Study of the Efficacy and Safety of Ruxolitinib to Treat COVID-19 Pneumonia](https://clinicaltrials.gov/ct2/show/NCT04331665?term=ruxolitinib&cond=Covid19&draw=2&rank=8) | COVID-19  Pneumonia | No Results Available | Princess Margaret Cancer Centre Toronto, Ontario, Canada | clinicaltrials.gov |
| 9 | Not yet recruiting | [Efficacy of Intravenous Anakinra and Ruxolitinib During COVID-19 Inflammation (JAKINCOV)](https://clinicaltrials.gov/ct2/show/NCT04366232?term=ruxolitinib&cond=Covid19&draw=2&rank=9) | Covid-19 | No Results Available | AP-HM, Hôpital de la Conception Marseille, Bouches-du-Rhône, France  (and 2 more...) | clinicaltrials.gov |
| 10 | Recruiting | [Phase 3 Randomized, Double-blind, Placebo-controlled Multi-center Study to Assess the Efficacy and Safety of Ruxolitinib in Patients With COVID-19 Associated Cytokine Storm (RUXCOVID)](https://clinicaltrials.gov/ct2/show/NCT04362137?term=ruxolitinib&cond=Covid19&draw=2&rank=10) | Cytokine Storm (Covid-19) | No Results Available | Novartis Investigative Site Aurora, Colorado, United States  (and 40 more...) | clinicaltrials.gov |
| 11 | Not yet recruiting | [Ruxolitinib for the Treatment of Acute Respiratory Distress Syndrome in Patients With COVID-19 Infection](https://clinicaltrials.gov/ct2/show/NCT04361903?term=ruxolitinib&cond=Covid19&draw=2&rank=11) | Severe Acute Respiratory Syndrome Coronavirus 2 | No Results Available |  | clinicaltrials.gov |
| 12 | Recruiting | [Ruxolitinib in Covid-19 Patients With Defined Hyperinflammation](https://clinicaltrials.gov/ct2/show/NCT04338958?term=ruxolitinib&cond=Covid19&draw=2&rank=12) | Covid-19 | No Results Available | Universitätsmedizin Göttingen - Klinik für Hämatologie und Onkologie Göttingen, Germany  (and 5 more...) | clinicaltrials.gov |
| 13 | Not yet recruiting | [A Trial Using ANAKINRA, TOCILIZUMAB Alone or in Association With RUXOLITINIB in Severe Stage 2b and 3 of COVID19-associated Disease](https://clinicaltrials.gov/ct2/show/NCT04424056?term=ruxolitinib&cond=Covid19&draw=2&rank=13) | Covid19 | No Results Available | Assistance Publique Hôpitaux de Marseille Marseille, France | clinicaltrials.gov |
| 14 | Not yet recruiting | [Therapeutic Plasma Exchange Alone or in Combination With Ruxolitinib in COVID-19 Associated CRS](https://clinicaltrials.gov/ct2/show/NCT04374149?term=ruxolitinib&cond=Covid19&draw=2&rank=14) | Cytokine Release Syndrome  COVID19 | No Results Available |  | clinicaltrials.gov |
| 15 | Recruiting | [Ruxolitinib for Acute Respiratory Disorder Syndrome Due to COVID-19](https://clinicaltrials.gov/ct2/show/NCT04477993?term=ruxolitinib&cond=Covid19&draw=2&rank=15) | Severe Acute Respiratory Syndrome Coronavirus 2  SARS-CoV2 | No Results Available | Hospital das Clínicas Sao Paulo, Brazil | clinicaltrials.gov |
| 16 | Recruiting | [Ruxolitinib for Treatment of Covid-19 Induced Lung Injury ARDS](https://clinicaltrials.gov/ct2/show/NCT04359290?term=ruxolitinib&cond=Covid19&draw=2&rank=16) | ARDS, Human  COVID | No Results Available | Andreas Neubauer Marburg, Germany | clinicaltrials.gov |
| 17 | Recruiting | [Study of Ruxolitinib Plus Simvastatin in the Prevention and Treatment of Respiratory Failure of COVID-19.](https://clinicaltrials.gov/ct2/show/NCT04348695?term=ruxolitinib&cond=Covid19&draw=2&rank=17) | Coronavirus Infection | No Results Available | Hospital Universitario Madrid Sanchinarro Madrid, Spain | clinicaltrials.gov |
| 18 | Recruiting | [COLchicine Versus Ruxolitinib and Secukinumab In Open Prospective Randomized Trial](https://clinicaltrials.gov/ct2/show/NCT04403243?term=ruxolitinib&cond=Covid19&draw=2&rank=18) | COVID 19 | No Results Available | Lomonosov Moscow State University Medical Research and Educational Center Moscow, Moscow Region, Russian Federation | clinicaltrials.gov |
| 19 | Recruiting | [A Systems Approach to Predict the Outcome of SARS-CoV-2 in the Population of a City; COVID-19](https://clinicaltrials.gov/ct2/show/NCT04351503?term=ruxolitinib&cond=Covid19&draw=2&rank=19) | SARS Coronavirus (SARS-CoV-2) Infection | No Results Available | Viollier AG Allschwil, Switzerland  (and 5 more...) | clinicaltrials.gov |
| 20 | Recruiting | [Pharmacokinetics, Pharmacodynamics, and Safety Profile of Understudied Drugs Administered to Children Per Standard of Care (POPS)](https://clinicaltrials.gov/ct2/show/NCT04278404?term=ruxolitinib&cond=Covid19&draw=2&rank=20) | Coronavirus Infection (COVID-19)  Pulmonary Arterial Hypertension  Urinary Tract Infections in Children  (and 22 more...) | No Results Available | Arkansas Children's Hospital Research Institute Little Rock, Arkansas, United States  (and 34 more...) | clinicaltrials.gov |
| 21 | Ongoing | MATIS: Phase 2/3, Randomised, Open-Label, Single-Site, Multi-Arm Trial of Ruxolitinib Plus Best Supportive Treatment (BST) versus Fostamatinib Plus BST versus BST for COVID-19 pneumonia | COVID-19 pneumonia | No Results Available | Great Britain | clinicaltrialsregister.eu  Sponsor Name:Joint Research Compliance Office  EudraCT Number: 2020-001750-22  Sponsor Protocol Number: 20HH5926  Start Date: 2020-07-09 |
| 22 | Ongoing | Ruxolitinib therapy to Avoid Ventilation and improve outcome for deteriorating COVID-19 patiENts - RAVEN | Severe COVID-19 infection with risk of need for mechanical ventilation. | No Results Available | Great Britain | clinicaltrialsregister.eu  Sponsor Name: Guy’s and St Thomas' NHS Foundation Trust  EudraCT Number: 2020-001777-71  Sponsor Protocol Number: RAVEN  Start Date: 2020-05-06 |
| 23 | Ongoing | An open prospective randomized therapeutic trial using ANAKINRA or TOCILIZUMAB alone or in combination with RUXOLITINIB in severe stage 2b and 3 COVID-19 disease | COVID-19-associated disease | No Results Available | France | clinicaltrialsregister.eu  Sponsor Name:Assistance Publique Hôpitaux de Marseille  EudraCT Number: 2020-001754-21  Sponsor Protocol Number: 2020-23  Start Date: 2020-05-19 |
| 24 | Ongoing | Randomized phase II clinical trial of ruxolitinib plus simvastatin in the prevention and treatment of respiratory failure of COVID-19.Ruxo-Sim-20 clinical trial. | COVID19 | No Results Available | Spain | clinicaltrialsregister.eu  Sponsor Name:Fundación de Investigación HM Hospitales  EudraCT Number: 2020-001405-23  Sponsor Protocol Number: Ruxo-Sim-20  Start Date: 2020-04-11 |
